# Dopamine receptor agonists mechanism of actions on glucose lowering and their connections with prolactin actions

**DOI:** 10.3389/fcdhc.2023.935872

**Published:** 2023-03-09

**Authors:** Hung-Yu Chien, Su-Mei Chen, Wan-Chun Li

**Affiliations:** ^1^ Division of Endocrinology and Metabolism, Department of Internal Medicine, Taipei City Hospital, Taipei, Taiwan; ^2^ Division of Nuclear Medicine, Department of Internal Medicine, Taipei City Hospital, Taipei, Taiwan; ^3^ Institute of Oral Biology, School of Dentistry, National Yang Ming Chiao Tung University, Taipei, Taiwan

**Keywords:** central insulin resistance, dopamine receptor agonists, prolactin, insulin resistance, insulin secretion

## Abstract

Robust experiment evidence suggests that prolactin can enhance beta-cell proliferation and increase insulin secretion and sensitivity. Apart from acting as an endocrine hormone, it also function as an adipokine and act on adipocytes to modulate adipogenesis, lipid metabolism and inflammation. Several cross-sectional epidemiologic studies consistently showed that circulating prolactin levels positive correlated with increased insulin sensitivity, lower glucose and lipid levels, and lower prevalence of T2D and metabolic syndrome. Bromocriptine, a dopamine receptor agonist used to treat prolactinoma, is approved by Food and Drug Administration for treatment in type 2 diabetes mellitus since 2009. Prolactin lowering suppress insulin secretion and decrease insulin sensitivity, therefore dopamine receptor agonists which act at the pituitary to lower serum prolactin levels are expected to impair glucose tolerance. Making it more complicating, studies exploring the glucose-lowering mechanism of bromocriptine and cabergoline have resulted in contradictory results; while some demonstrated actions independently on prolactin status, others showed glucose lowering partly explained by prolactin level. Previous studies showed that a moderate increase in central intraventricular prolactin levels stimulates hypothalamic dopamine with a decreased serum prolactin level and improved glucose metabolism. Additionally, sharp wave-ripples from the hippocampus modulates peripheral glucose level within 10 minutes, providing evidence for a mechanistic link between hypothalamus and blood glucose control. Central insulin in the mesolimbic system have been shown to suppress dopamine levels thus comprising a feedback control loop. Central dopamine and prolactin levels plays a key role in the glucose homeostasis control, and their dysregulation could lead to the pathognomonic central insulin resistance depicted in the “ominous octet”. This review aims to provide an in-depth discussion on the glucose-lowering mechanism of dopamine receptor agonists and on the diverse prolactin and dopamine actions on metabolism targets.

## 1 Introduction

Type 2 diabetes mellitus(T2D) is both an endocrine and metabolic disease and the prevalence is increasing exponentially worldwide (1). The pathogenesis of T2D have expanded from dysfunctions of “ominous triumvirate” (beta-cell dysfunction and insulin resistance in muscle, liver) proposed since 1987 by Defronzo to “ominous octet”. The octet includes the fat cell (accelerated lipolysis), gastrointestinal tract (incretin deficiency/resistance), α-cell (hyperglucagonemia), kidney (increased glucose reabsorption), and brain (insulin resistance) (2). T2D develops when the dynamic balance between beta cell (β-cell) proliferation cannot keep up with the rate of β-cell apoptosis. At a point when overt impaired glucose sensing and insufficient β-cell mass ensues, insulin secretion fails to compensate for increased insulin resistance then impaired glucose tolerance and T2D develops (3, 4). In humans β-cell proliferation is exceedingly low, as contrasted to rodents, but is able to expand rapidly under specific situations, such as pregnancy, when prolactin(PRL) and placental lactogen play a pivotal role in stimulating β-cells expansion (5).

**Figure 1:**
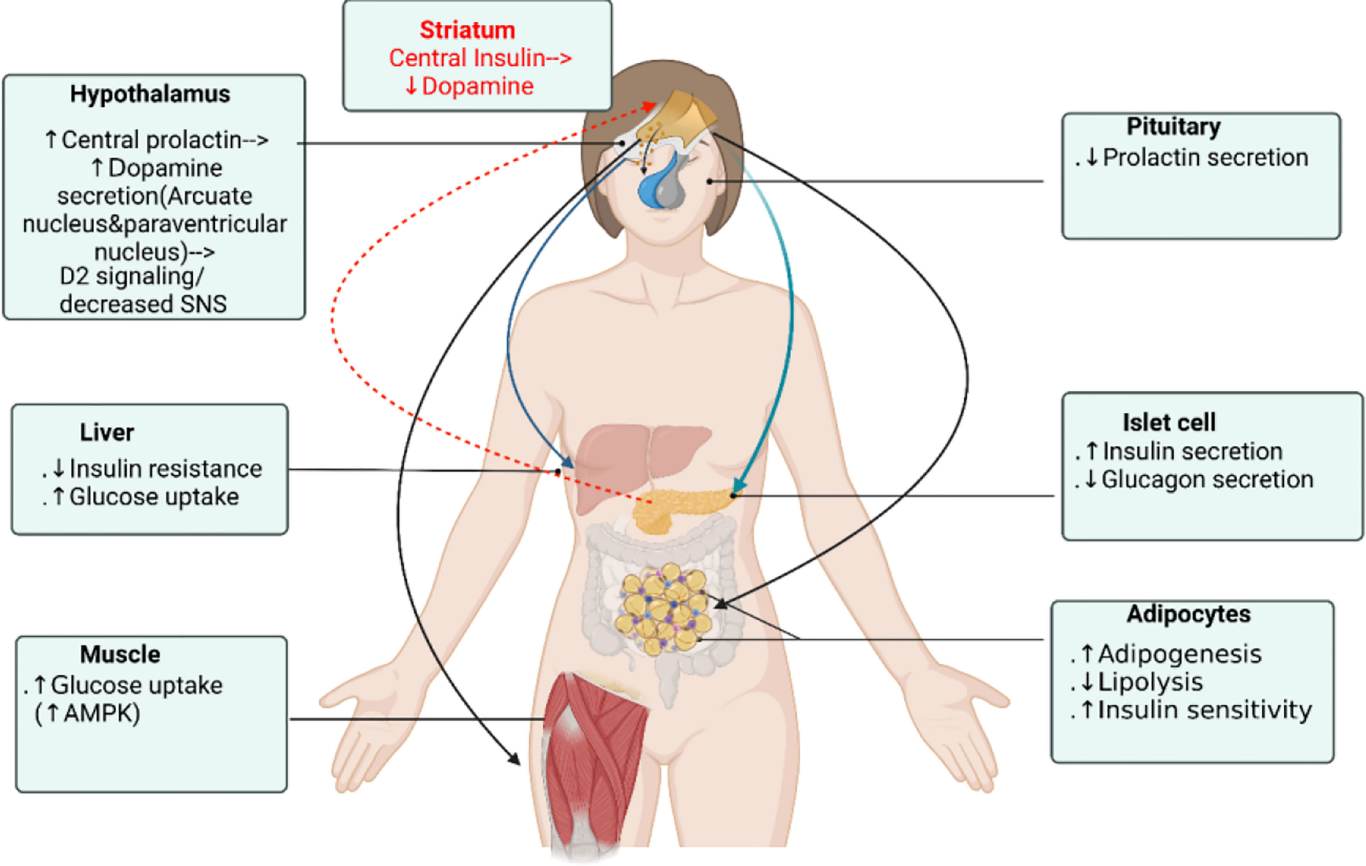
Dopamine receptor agonist glucose-lowering actions on different organs. Dopamine agonists affect glucose homeostasis by 1) stimulating central prolactin that increase ventral medial and arcuate hypothalamic dopamine that act through 2) dopaminergic/decreased sympathetic activity signaling which 3) directly act on islet to stimulate insulin secretion and on adipocyte, hepatocyte, skeletal muscle D2R to modulate their metabolic effects, not by the serum prolactin level. Reciprocally, central insulin suppresses dopamine level in the ventral striatum and mesolimbic dopamine system. Figure created using BioRender (https://biorender.com/).

Prolactin (PRL) is a polypeptide hormone that is mainly synthesized and secreted by lactotroph cells of the anterior pituitary gland. In humans, PRL is synthesized in the anterior pituitary but also in the hypothalamus, decidua, endometrium, breast, prostate, lymphocytes, leukocytes and adipocytes (6, 7). The well-known role attributed to PRL is to activate the differentiation and proliferation of the mammary cells necessary for lactation. However, as the prolactin receptor (PRLR) is expressed in various tissues and cells (including pancreatic islets, adipocytes, endometrium and the prostate), PRL also modulates and regulates many other physiological processes including metabolism, growth, reproduction, immune regulation, angiogenesis and osmoregulation (8–10). Compelling experimental evidence shows that PRL acts on β-cells to enhance β-cell proliferation and increase insulin secretion and sensitivity (11, 12). It is also an adipokine and act on adipocytes to regulate adipogenesis, lipid metabolism and inflammation (13). Several cross-sectional epidemiologic studies consistently showed that circulating PRL levels positive correlated with increased insulin sensitivity, lower glucose and lipid levels, and lower prevalence of T2D and metabolic syndrome (14, 15). On the other hand, PRL excess in supraphysiologic levels result in increase of HOMA-IR(surrogate index of insulin resistance) (16, 17), in both obese and lean patients. PRL excess induces leptin resistance and inhibition of dopaminergic tone which stimulates food intake and in turn may lead to increased body weight and adiposity in patients with hyperprolactinemia (18–20). Macotela et al. recently defined “homeostatic functionally increased transient prolactinemia” as their study showed the beneficial outcome of PRL on metabolism depends on circulating PRL levels kept within 7 and 100 ng/mL (21). Dopamine agonists bromocriptine and cabergoline have long been the treatment of choice for patients with prolactinomas owing to t+heir good therapeutic response (22–25). In patients with prolactinomas treated with bromocriptine and cabergoline, glucose metabolism and insulin sensitivity significantly improved in most patients but only some patients showed body weight reduction (26, 27). Moreover, in both type 2 diabetic and non-diabetic subjects, bromocriptine treatment lowered glycated hemoglobin (HbA1c), fasting plasma glucose and reduced body weight (25, 28) and had been approved by the Food and Drug Administration for treatment in type 2 diabetes mellitus since 2009. Since PRL lowering mechanistically leads to suppressed insulin secretion and decreased insulin sensitivity, dopamine agonist should be expected to worsen glucose tolerance. Making it even more complicating, studies exploring the glucose-lowering mechanism of bromocriptine and cabergoline have resulted in contradictory results, while some demonstrated significant improvement in glucose metabolism independently on PRL status (25, 29, 30), others showed glucose-lowering in T2DM patients partly explained by PRL level (31). Studies by Park et al. showed that a moderate increase in central intraventricular prolactin (1ug/h chronic infusion into lateral ventricle) levels elevated hypothalamic dopamine levels with a decreased serum PRL level and improved glucose metabolism in diabetic rats (32). It has been shown that animal and human pancreatic β-cells (33) and human adipocytes (34) all express dopamine receptors type 2 (D2R), so both dopamine and PRL acts at peripheral level to modulate β-cells, adipocytes, skeletal muscle and hepatocytes functions. Additionally, recently shown clusters of sharp wave-ripples from the hippocampus lowered peripheral glucose concentrations within 10 minutes by suppressing activity of the lateral septum in rats, which is the major conduit between the hippocampus and the hypothalamus (35). Hippocampal outputs affect endocrine and hormonal systems through hypothalamic hormone-releasing neurons or autonomic control of peripheral organs. This provides a mechanism unrevealing direct central neurohormonal control on β-cell insulin secretion. Central insulin in the mesolimbic system have been shown to suppress dopamine levels thus comprising a feedback control loop (36). It appears that dopamine agonists affect glucose homeostasis by 1) stimulating central PRL that increase ventral medial and arcuate hypothalamic dopamine which act through 2) increased dopaminergic/decreased sympathetic activity signaling. These signal 3) directly act on islet and adipocytes, hepatocytes, skeletal muscle D2R to modulate their metabolic effects and reciprocally insulin feedback inhibits the mesolimbic dopamine system (**Figure 1**). This review aims to provide an in-depth discussion on the glucose-lowering mechanism of dopamine receptor agonists and on the diverse prolactin and dopamine actions on metabolism targets including β-cells, adipocytes, hepatocytes, skeletal muscle and central nervous system (CNS).

### 1.1 Peripheral effects of PRL within the physiologic range

#### 1.1.1 Peripheral PRL effect on β (beta)-cell

Both human islet cell lines and *in vitro* rodent studies have demonstrated that adding PRL to isolated pancreatic islet enhanced insulin secretion and β-cell proliferation (11), and overexpression of PRL in β-cells resulted in inappropriately increased insulin synthesis and secretion, also enhancing β-cell proliferation. Similarly, overexpression of PL in β-cells resulted in fasting and postprandial hypoglycemia, marked elevated serum insulin levels, increased islet insulin granules and volume and increased β-cell numbers and proliferation (37). In contrast, studies in PRLR knockout (Prlr-) mice demonstrates the fundamental role of PRL and PL during pancreatic islet development. PRLR knockout (Prlr-) mice developed impaired glucose tolerance, decreased insulin secretion, a 20-35% reduction in islet insulin granules, reduced islet density and reduced β-cell mass. Prlr- mice also showed impaired glucose tolerance and blunted insulin secretory response comparing to their wild-type littermates (38).

PRLR is highly expressed on pancreatic β-cell. Both PRL and placental lactogen (PL) that rise during mid-pregnancy lactogenic surge enhance β-cell proliferation and glucose-stimulated insulin hypersecretion, act through PRLR (39, 40). It is well established that the β-cell trophic and metabolic modulation effect of prolactin is not limited to the period of pregnancy (41, 42). These studies provide strong evidence that PRL and PL can enhance β-cell insulin secretion and proliferation. Although numerous evidence showed that lactogenic hormones do regulate β-cell mass during pregnancy, their actions cannot fully explain all of the changes that appears in β-cell mass during gestation. Despite persistent elevated PRL and PL concentrations till the end of pregnancy and even towards the end of lactation, the β-cell proliferation peaks around mid-gestation and drops to a normal rate seen in non-pregnant state and an even lower rate by parturition, leading to rapid normalization of β-cell mass postpartum. Although β-cell numbers and mass increase about 1.4–2.4 times relative to normal (43) during mid-pregnancy lactogenic surge, in patients with gestational diabetes mellitus (GDM), insulin requirements often increase during the third trimester that coincides with the increase in insulin resistance to maintain good glycemic control. Also, insulin requirements double during gestational week 16 to week 37 in type1 DM, which is similar in magnitude to an increase in insulin resistance. Nieuwenhuizen et al. showed that rather than PRL, decreased insulin sensitivity per se during gestation plays a more important role in the regulation of β-cell proliferation (44).

#### 1.1.2 Peripheral PRL effect on adipocyte and insulin resistance

Adipose tissue is the largest organ in the human body and adipokines plays a pivotal role in metabolic and endocrine homeostasis. In obesity, accumulation of excessive fat is distributed either into existing adipocytes (hypertrophy) or into newly recruited adipocytes (hyperplasia) generated by adipogenesis from differentiation of pre-adipocytes. Adipose tissue expansion in the form of hyperplasia is generally regarded as a more metabolic healthy process (45, 46), whereas expansion in the form of excessive adipocyte hypertrophy leads to metabolic disruption and associated with insulin resistance and metabolic syndrome (47). PRL have been shown to promote adipose differentiation and enhance adipocyte hyperplasia and may be a potential therapeutic target against insulin resistance and metabolic syndrome. Adipocytes have been shown to express PRLR (48) and D2R (34), and both PRL and dopamine are produced by human adipose tissue (49). A large body of literature indicates that PRL participates in many aspects of adipose tissue actions, including adipogenesis (50, 51), metabolic enzyme activity, lipolysis, and release of adipokines such as leptin, adiponectin, and inflammatory cytokines such as Il-1, IL-6 and monocyte chemoattractant protein 1 (52, 53). Analogous to the pancreatic islet, adipocytes adapt during pregnancy and lactation, when PRL values may exceed 200 μg/L, including neural (dopaminergic) changes that increase appetite, food intake, and redistribute nutrients, lipid storage from abdominal tissues to mammary glands (54). Physiological levels of PRL is about 10-25 mg/dL in women and 10-20 mg/dL in men and may reach up to 90 mg/dl after exercise, eating, sexual intercourse, general anesthesia, surgical operations, and stress. Recent large cohort studies established that, within normal range, low serum PRL is associated with metabolic syndrome and obesity in children (37), and higher prevalence of impaired glucose intolerance, increased insulin resistance, polycystic ovary syndrome (PCOS), nonalcoholic fatty liver disease (NAFLD), and increased risks of new onset T2D in both men and women (11–18).

In concordance with epidemiologic studies, circulating levels of PRL are reduced in the ob/ob and db/db obese and diabetes mouse models, and in streptozocin-induced (STZ) T2DM rats. The 24 hour secretory pattern of plasma PRL levels is disrupted and reduced in high fat diet(HFD) rats (55), and Ruiz-Herrera et al. showed that raising plasma prolactin levels to 60-80 mg/dL equivalent to those reported in male rats in response to stress (56) counteracts the insulin resistance and adipose tissue dysfunction (57).

On the other hand, PRL is essential for adipose tissue differentiation and Prlr- mice with HFD demonstrated reduced adipocyte hyperplasia, increased adipocyte hypertrophy and increased insulin resistance (Table 1). Prlr- mice showed reduced fat and body weight after 16 weeks which is more prominent in females than males composed with a 29% decrease in fat mass, reduced leptin level and an even more pronounced 49% decrease in VAT (58). The decrease in fat depot is due to a reduced number of adipocytes (59). Aside from the effect on white adipose tissue, PRL have also been demonstrated to play a fundamental role in brown adipose tissue (BAT) differentiation. PRL promotes the generation and expansion of BAT in newborn rodents. BAT mass is significantly reduced in neonate Prlr- mice in comparison to their wild-type littermates with reduced brown adipocyte triglyceride content (60).

**Table 1:** Differential effects of various dopamine agonists and antagonists on PRL levels and glucose tolerance.

Dopamine receptor antagonists	Serum prolactin	Central prolactin	Glucose tolerance
metoclopropamide	⇧	⇧	→
domperidone	⇧	⇧	→
risperidone	⇧	⇧	→
amilsulpride	⇧	⇧	→
olanzapine	⇧	→	↓
Dopamine receptor agonists			
bromocriptine	↓	⇧	⇧
Cabergoline	↓	⇧	⇧

Solid arrows, stimulates; Dashed arrows, inhibits.

### 1.2 Central effects of PRL within the physiologic range

#### 1.2.1 Central PRL effect on β (beta)-cell

**Figure 2:**
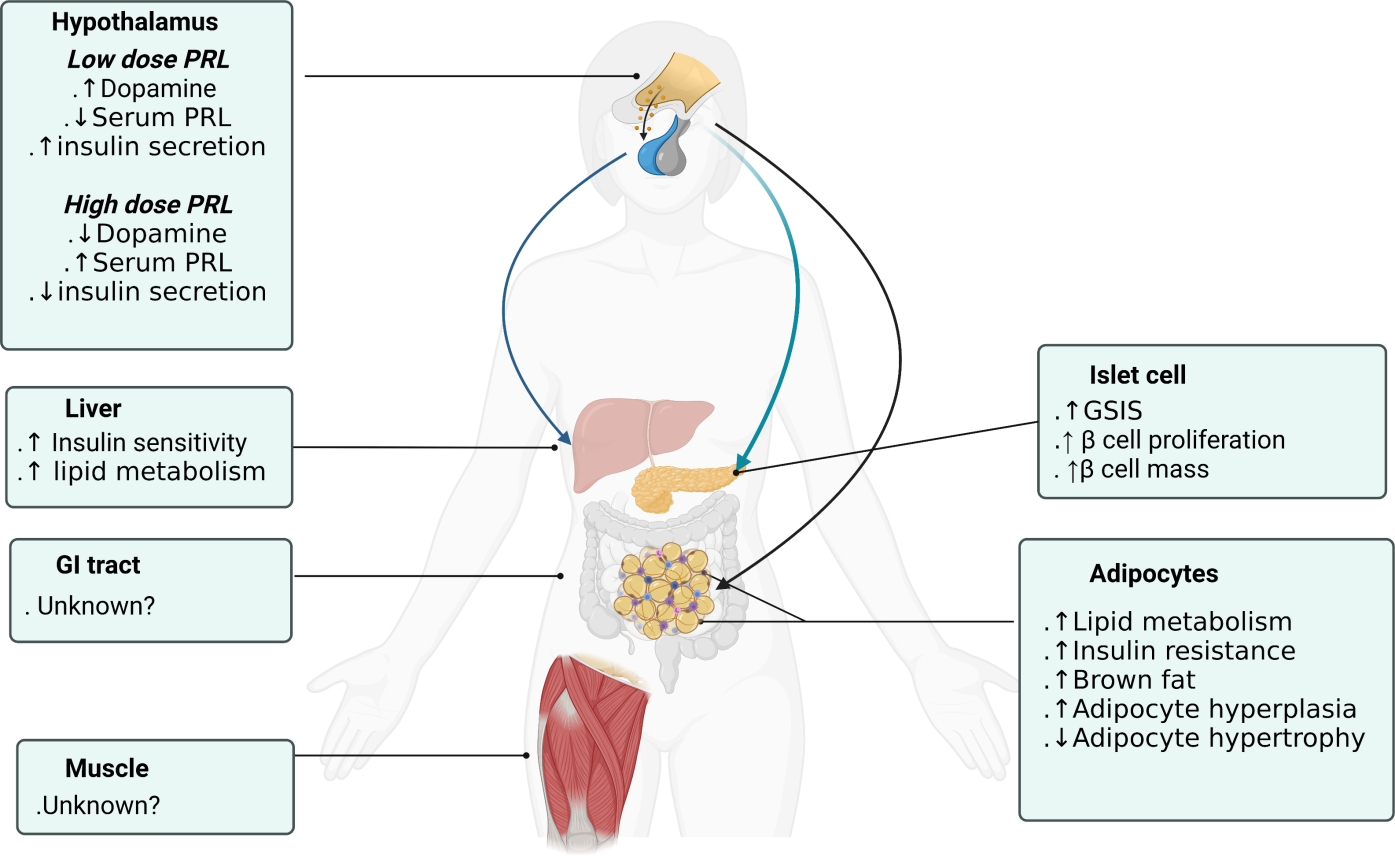
Prolactin actions on different organs to promote metabolic homeostasis. Figure created using BioRender (https://biorender.com/).

Central actions of PRL are very diverse from its peripheral actions. Centrally administered low-dose PRL (0.1ug/h infusion) increased hypothalamus dopamine levels, decreased serum prolactin and improved insulin secretory capacity and sensitivity. In contrast, high-dose central PRL (1ug/h infusion) failed to increase hypothalamic dopamine, increased serum prolactin and decreased insulin secretion and sensitivity. It is suggested that the short-loop regulation of prolactin secretion by dopamine is desensitized and disrupted with continuous high-dose PRL stimulation. PRL acts on specific dopamine neurons in the hypothalamic arcuate nucleus and periventricular nucleus that increase dopamine secretion which tuberoinfundibular pathway inhibit prolactin release from the pituitary gland (61) (**Figure 2**). These dopaminergic neurons not only modulate PRL secretion, but also regulate the orexigenic and anorexigenic neurons within the arcuate nucleus (62). Central insulin in the dorsal striatum has been shown to reciprocally suppress dopamine levels (36) comprising a feedback control loop. In addition, low-PRL decreased body weight and epididymal fat pads and improved insulin sensitivity in T2DM rats which is consistent with dopamine receptor agonist actions described in paragraph 2.3. Only low-dose PRL stimulated first-phase insulin secretion and improved insulin sensitivity. Despite no increase in serum PRL level, both low and high central PRL dosages enhanced β-cell expansion suggesting that central prolactin could directly regulate β-cell function and proliferation regardless of serum prolactin levels (32). In summary, central prolactin regulates insulin secretion and glucose metabolism through central dopamine release and D2 receptor/sympathetic activity signaling (63). And β-cell stimulatory effects depend more on direct neural dopaminergic signal rather than on serum prolactin level.

#### 1.2.2 Central PRL effect on adipocyte and insulin resistance

No direct effect of central PRL on adipocytes have been discovered. However, dopamine D2/3 receptor inhibition at the ventral striatum results in diminished insulin sensitivity (64) and decreased mesolimbic dopamine activity leads to insulin resistance (65).

#### 1.2.3 Central PRL effect on appetite regulation and hypothalamus

Within normal concentrations, PRL is not considered as a major factor in the occurrence of obesity. As discussed in paragraph 2.1.2, locally produced PRL is involved in the regulation of human adiposity and dysregulation leads to perpetuation of obesity. PRLR shares the same signaling pathways with leptin which is well known involved in regulation of appetite, metabolism and energy balance. In rodent models, increased serum PRL levels provoke leptin resistance that stimulates food intake and results in increased body weight and adiposity (66–68). During pregnancy and lactation, plasma leptin levels reduce by 40% for metabolic adaptions when high PRL levels, leptin resistance, hyperphagia (69), increased body weight coincide to facilitate nutrient shift to offspring (70). Some studies suggested that PRLR activation enhanced expression of suppressor-of-cytokine signaling (SOCS) proteins at hypothalamus including arcuate nucleus, medial preoptic area and the nucleus of the solitary tract which inhibited shared downstream signaling pathways of leptin receptor and leads to increased appetite and body weight (71). In contrast, Prlr- mice treated with leptin antagonist showed Prlr- mice responded to leptin antagonist similarly to the control mice, indicating no interaction between the actions of the two hormones (54). Such discrepancies may occur since insulin co-expression at PRLR is necessary for prolactin mediated effects (72) and central insulin suppresses dopamine levels in the ventral striatum (36). Body weight and adiposity are regulated and coordinated by a variety of factors including brain appetite signals, neuroendocrine signals, nutritional input and energy expenditure. In summary, whether elevated PRL levels in prolactinoma patients results in hyperphagia and overweight is controversial, since lowering PRL levels with D2R agonists do not always associate with reduced body weight and can even result in body weight gain (43, 73), implicating other factors such as dopaminergic signals may play a more dominant role.

### 1.3 Central effects of PRL excess and its correction with dopamine agonists

Prolactinomas or other causes of pathological hyperprolactinemia are associated with an increased risk of T2D. Mechanistically, pathological hyperprolactinemia has a negative effect on post-prandial insulin sensitivity (69). Despite the afore mentioned action of PRL to stimulate insulin secretion and increase insulin sensitivity, pathologic elevated levels of PRL (>70ng/ml) leads to increased insulin resistance (17, 74). Hyperinsulinemic-euglycemic clamp studies demonstrated post-prandial hyperinsulinemia and a significant decrease in peripheral post-prandial insulin sensitivity in both hyperprolactinemia obese and non-obese subjects (27, 69). In addition, hyperprolactinemia attenuate adiponectin expression in adipose tissue (75), which also decrease insulin sensitivity (76, 77).

Some frequently used dopamine receptor antagonists, such as metoclopramide, domperidone and risperidone, elevates both serum and central (cerebrospinal fluid) PRL levels and by doing so do not disturb energy and glucose metabolism, while other dopamine antagonists, such as olanzapine, induce impaired glucose tolerance and obesity since they do not increase central prolactin levels (78). And another dopamine antagonist, amisulpiride, a preferential antagonist at presynaptic D2/D3 receptors that increase dopaminergic transmission reduces glucose levels in diet-induced obese mice (79) and human (80). Studies with chronic hyperprolactinemia evoked by selective D2R knockout in lactotrophs leads to early glucose intolerance, hyperinsulinemia and subsequent liver steatosis and marked adiposity (81). Whereas studies with global D2R knockout mice develops impaired glucose tolerance, indicating pancreatic D2R participate in the effect of dopamine agonists (82). Recently, Aslanoglou showed that pancreatic hormone (insulin, and glucagon) secretion modulated by dopamine receptor agonists is dependent on D2R expression levels and D2 antagonist significantly increase glucagon as well as insulin secretion in human islets and treatment with D2 antagonists, such as olanzapine, frequently leads to impaired glucose homeostasis (83). Of note, hyperprolactinemia does not disturb glucose homeostasis in global D2R knockout mice (84), indicating that central dopaminergic system is crucial for the metabolic effects of PRL.

On the other hand, dopamine receptor agonists suppressed glucose-stimulated insulin secretion both in rats (85) and in patients with Parkinson’s disease (PD) (86). In prolactinoma patients treated with bromocriptine and cabergoline, glucose metabolism improved with increased insulin sensitivity. Increased insulin sensitivity correlated with bromocriptine dose measured by euglycemic hyperinsulinemic clamp. In studies treated with cabergoline, reduction in fasting insulin and HOMA-IR also correlated with cabergoline dose but not to body weight and BMI, suggesting that directly cabergoline could modulate insulin secretion and sensitivity. These actions are not limited to hyperprolactinemia state and have also been demonstrated in both diabetic and non-diabetic normoprolactinemia subjects (24, 25). Bromocriptine therapy in T2D subjects poorly controlled on high-dose insulin therapy resulted in improved glycemic control and meal tolerance while reducing insulin requirement (87).

Since PRL lowering action is the major mechanism of bromocriptine in prolactinoma treatment, it is also reasonable it contributed to a glucose-lowering effect. However, Framnes-DeBoer et al. demonstrated that bromocriptine improves glucose metabolism in prolactin-deficient, MC4R-deficient, and circadian disrupted mice (88) indicating that the glucose-lowering effect is independent of prolactin, MC4R, or circadian rhythms. In contrast, Furigo et al. using PRL antagonist(G129R-hPrl) treatment showed that bromocriptine effects on glucose metabolism is partly associated with blocked PRL actions, but it appeared only in female mice (31). While the exact glucose-lowering mechanism of bromocriptine remains undefined, it is postulated that bromocriptine may act by decreasing sympathetic activity (89). Decreased dopaminergic signals to the suprachiasmatic nucleus results in impaired glucose intolerance and increased insulin resistance in animal models (90). Studies have shown bromocriptine directly increases brain dopamine levels, most likely at the ventromedial nucleus of hypothalamus that lowers sympathetic activity, which leads to improved glucose homeostasis (91). Low concentrations of bromocriptine, dopamine have been shown to stimulate prolactin release from cultured pituitary tumor cell line GH4ZR7 cells while high concentrations inhibited the release (92). Haloperidol, a D2 receptor antagonist, blocked the inhibitory action, but was unable to block the dopamine-induced stimulatory action (93). Central PRL acts on specific dopaminergic neurons in the arcuate nucleus and paraventricular nucleus of the hypothalamus to increase dopamine secretion (61). Islet insulin secretion is mainly regulated by glucose uptake by glucose transporter-2 (GLUT-2), but it is also controlled by the central nervous system through parasympathetic and sympathetic nerves (94). Recently shown clusters of sharp wave-ripples from the hippocampus lowered peripheral glucose concentrations within 10 minutes by suppressing activity of the lateral septum, which is the major conduit between the hippocampus and the hypothalamus (35). It is suggested that hippocampal outputs affect neuroendocrine systems through hypothalamic hormone-releasing neurons or autonomic control of peripheral organs. Studies in humans showed that central insulin directly suppresses dopamine levels in the ventral striatum (36). And dopamine D2/3 receptor inhibition at the ventral striatum results in diminished insulin sensitivity (64) and in concert decreased mesolimbic dopamine activity leads to insulin resistance (65). In summary, central prolactin and dopamine levels work in coordination to control peripheral insulin secretion, and central insulin levels in turn feedbacks on mesolimbic dopamine system comprising a closed control system.

### 1.4 Peripheral effects of PRL excess and its correction with dopamine agonists (adipose tissue, skeletal muscle, hepatocyte, insulin resistance)

Ruiz-Herrera et al. showed that raising plasma prolactin levels to 60-80 mg/dL equivalent to those reported in response to stress (56) counteracts the insulin resistance and adipose tissue dysfunction in high fat diet(HFD) rats (57). PRL by increasing circulating adiponectin shows beneficiary effect on metabolism function, and these actions depends on circulating PRL levels kept at a relatively high concentration, but within a physiological range (within 7 and 100 ng/mL), defined as “homeostatic functionally increased transient prolactinemia” by Macotela (21). Mechanistically, PRL treatment in obese, insulin-resistant rats promotes adipocyte hyperplasia in both visceral adipose tissue (VAT) and subcutaneous adipose tissue (SAT), as well as reducing adipocyte hypertrophy in VAT. PRL increases adiponectin levels by Xbp1s which induces adipogenesis *via* the transcriptional activation of CEBP alpha and PPAR gamma (95). In both lean and obese mice, PRL increases adiponectin levels that improved glucose tolerance and insulin sensitivity (96).

In humans, consistent with animal models, Ruiz et al. showed that PRL values are higher in insulin sensitive males than insulin resistant males, regardless of their body mass index(BMI), and that PRL levels are positively associated with adiponectin levels and with the adipocyte fitness makers expressions, including Adiponectin, C1Q And Collagen Domain Containing (ADIPOQ), peroxisome proliferator-activated receptors gamma(PPAR gamma) and glucose transporter 4(GLUT4). In addition, ADIPOQ, PPAR gamma and GLUT4 expression in SAT positively correlated with adipocytes PRL synthesis (57). Recently, Ponce et al. extended these findings showing that circulating PRL levels are associated with VAT adipocyte size in humans (97). Patients with low circulating PRL levels have hypertrophic visceral adipocytes and are associated with increased insulin resistance. Consistent with this, visceral adipocytes from low PRL levels show higher PRLR expression than their subcutaneous counterparts (48). Patients with low circulating PRL have four-fold higher proportion of large adipocytes in only VAT but not SAT comparing to patients with high circulating PRL levels. In contrast, when insulin sensitive and insulin resistant subjects are compared, insulin resistant subjects have an increased proportion of large adipocytes in both VAT and SAT. Consistent with this, visceral adipocytes from insulin resistant subjects show lower PRLR expression and lower expression of adipocyte fitness makers ADIPOQ, PPAR gamma and GLUT4. Insulin resistance together with lower circulating PRL levels may act synergistically to impair beneficial metabolic action of PRL in adipocytes, and thereby exaggerate adipocyte hypertrophy and dysfunction (57). Verboven et al. also showed that adipocyte hypertrophy in VAT but not SAT is associated with increased inflammation and increased accumulation of pro-inflammatory immune cells (98). PRL treatment demonstrate anti-inflammatory effect in adipocytes and increases adiponectin serum levels and decreases IL-1β and monocyte chemoattractant protein 1(MCP 1) (57). However, it appears that exceeding a level of PRL (250 ng/mL) lowers systemic adiponectin values and negatively affect insulin sensitivity in adipocytes cell culture (76) and transgenic female (but not male) mice overexpressing PRL (74), which is discussed in paragraph 2.3. PRL by increasing circulating adiponectin shows beneficiary effect on metabolism function when kept in a range within 7 and 100 ng/mL, defined as “homeostatic functionally increased transient prolactinemia” by Macotela (21). Pathologic elevated levels of PRL (>70ng/ml) leads to increased insulin resistance (17, 74). Hyperinsulinemic-euglycemic clamp studies demonstrated post-prandial hyperinsulinemia and a significant decrease in peripheral post-prandial insulin sensitivity in both hyperprolactinemia obese and non-obese subjects (27, 69). In addition, hyperprolactinemia attenuate adiponectin expression in adipose tissue (75), which also decrease insulin sensitivity (76, 77). PRL and PRLR are related to fat metabolism and promote insulin resistance by activating the Janus kinase 2/signal transducer and activator of transcription 5 (JAK2/STAT5) signaling pathway and increasing triglyceride deposition (99).

Tavares et al. showed peripheral action of bromocriptine directly acts on dopamine receptors at the hepatocytes, skeletal muscle and adipocytes which activates adenosine monophosphate kinase (AMPK) phosphorylation to enhance glucose uptake and modulates lipid metabolism–related proteins in the adipose tissue (100). Bromocriptine treatment in diabetic rats upregulates dopamine receptors at adipose tissue and the liver resulting in higher insulin sensitivity and catabolic function (101).

Taken together, either locally produced PRL (autocrine, paracrine) or circulating PRL (endocrine) plays a principal role to modulate healthy accumulation of excessive fat in adipocytes. PRL promotes PPAR gamma, CEBP alpha and Xbp1s expression to favor adipogenesis, indicating PRL could directly alter adipocyte differentiation and preferentially distribute excessive fat into new adipocytes (increasing adipocyte hyperplasia) and also attenuate adipocyte hypertrophy and adipocyte inflammation (57). PRL are produced and secreted from human adipose tissue and PRL levels in obese adipose tissue are much reduced over lean subjects, indicating diminished autocrine and paracrine actions of PRL in obesity. Exactly how adipose autocrine PRL actions is disrupted under very low and high circulating prolactin level and how they mutually affect another remains for further exploration. In fact, evidence also demonstrate that dopaminergic action is tightly involved in the regulation of peripheral glucose homeostasis and insulin sensitivity discussed in the previous paragraphs. In summary, PRL enhances insulin sensitivity and the facilitate the healthy expansion of adipose tissue. These metabolic favorable mechanisms become disrupted under insulin resistant and obesity conditions, which is associated with low circulating PRL levels and could be counteracted by increasing PRL levels.

### 1.5 Central and peripheral effects of hypoprolactinemia

Studies in PRLR knockout (Prlr-) mice demonstrates the fundamental role of PRL and PL during pancreatic islet development. PRLR knockout (Prlr-) mice developed impaired glucose tolerance, decreased insulin secretion, a 20-35% reduction in islet insulin granules, reduced islet density and reduced β-cell mass. Prlr- mice also showed impaired glucose tolerance and blunted insulin secretory response comparing to their wild-type littermates (38).

PRL is also essential for adipose tissue differentiation and Prlr- mice with HFD demonstrated reduced adipocyte hyperplasia, increased adipocyte hypertrophy and increased insulin resistance. Prlr- mice showed reduced fat and body weight after 16 weeks which is more prominent in females than males composed with a 29% decrease in fat mass, reduced leptin level and an even more pronounced 49% decrease in VAT (58). The decrease in fat depot is due to a reduced number of adipocytes (59). Aside from the effect on white adipose tissue, PRL have also been demonstrated to play a fundamental role in brown adipose tissue (BAT) differentiation. PRL promotes the generation and expansion of BAT in newborn rodents. BAT mass is significantly reduced in neonate Prlr- mice in comparison to their wild-type littermates with reduced brown adipocyte triglyceride content (60).

However, hypoprolactinemia or a defect in the PRLR is an extraordinary rare clinical condition, with approximately a few dozen cases reported in the literature since 1975. The main clinical findings were excessive amniotic fluid (polyhydramnios) and lactation failure (102). Mutations in the genes encoding PRL or PRLR has not been shown to develop any disorder in non-pregnant condition (103).

## 2 Conclusion

In the past decade, major progress has emerged in understanding the multifaceted role of prolactin in metabolic homeostasis but is still far from being well understood. With adipocytes, prolactin exerts endocrine and autocrine actions which modulates insulin sensitivity and facilitate the healthy expansion of adipose tissue. However, their regulatory actions become disrupted during obesity and insulin resistance. As on islet cells, prolactin enhance β-cell proliferation and insulin secretion, and these actions depends more on direct neural dopamine signal affecting local prolactin level rather than on serum prolactin level per se. Dopamine receptor agonists which lowers serum prolactin affect glucose homeostasis by 1) affecting central prolactin that increase hypothalamic ventral medial and arcuate nucleus dopamine then through 2) dopaminergic/decreased sympathetic activity signaling, which 3) directly act on islet to stimulate insulin secretion and on adipocyte, hepatocyte, skeletal muscle D2R to modulate their metabolic effects (**Figure 1**). And reciprocally insulin suppresses mesolimbic dopamine system that comprise a feedback loop. The central dopamine and prolactin levels plays a key role in the glucose homeostasis control, and their dysregulation could lead to the pathognomonic central insulin resistance depicted in the “ominous octet”.

Patients with T2D have a higher risk of developing PD and the presence of T2D is associated with greater PD severity and faster progression. Central prolactin and dopamine actions play crucial roles in both diseases. Robust evidence shows the effects of bromocriptine treatment in type 2 diabetes. This review puts forward the plausible glucose-lowering mechanism of dopamine receptor agonists, however more studies are needed to clarify the specific actions of central dopamine and prolactin and dopaminergic signaling in insulin-sensitive tissues which would identify potential therapeutic strategies for the treatment of metabolic disorders including type 2 diabetes and metabolic syndrome and even Parkinson’s disease.

## 3 Review criteria

References for this review were identified through searches of PubMed for articles published from January 1970, to October 2022, by use of the terms “prolactin”, “placental lactogens”, “prolactin receptors”, “dopamine”, “dopamine receptor agonist, antagonist”, “insulin”, “insulin resistance”, “hyperprolactinemia”, and “hypoprolactinemia” in combination with the term “diabetes”. Articles resulting from these searches and relevant references cited in those articles were reviewed.

## Author contributions

Conceptualization, H-YC. Investigation, H-YC and S-MC. Writing—original draft preparation, H-YC. Writing—review and editing, H-YC and S-MC. Supervision, H-YC and W-CL. All authors contributed to the article and approved the submitted version.
